# Socioeconomic, physical and mental health impacts of climate change among informal outdoor workers in sub-Saharan Africa: A scoping review protocol

**DOI:** 10.1371/journal.pone.0344943

**Published:** 2026-03-13

**Authors:** Sylvia Hagan, Kate Nyhan, Ernest Darkwah, Collins Badu Agyemang, Yaw Agyeman Boafo

**Affiliations:** 1 Department of Psychology, University of Ghana, Legon, Accra, Ghana; 2 MacMillan Center for International and Area Studies, Yale University, New Haven, Connecticut, United States of America; 3 Harvey Cushing/ John Hay Whitney Medical Library, Yale University, New Haven, Connecticut, United States of America; 4 Environmental Health Sciences, Yale School of Public Health, Yale University, New Haven, Connecticut, United States of America; 5 Centre for Climate Change and Sustainability Studies, University of Ghana, Legon, Accra, Ghana; Independent Medical Researcher and Writer, UNITED KINGDOM OF GREAT BRITAIN AND NORTHERN IRELAND

## Abstract

The informal economy plays a critical role in Sub-Saharan Africa (SSA), yet informal outdoor workers are disproportionately exposed to climate-related hazards. Existing reviews often merge formal and informal workers, limiting insight into the distinct vulnerabilities and outcomes experienced in informal outdoor work. This proposed scoping review seeks to synthesise evidence on the socioeconomic, physical, and mental health impacts and coping strategies of climate change among informal outdoor workers in SSA. We will search Medline, Global Health, Embase, Scopus, Lens, PsycINFO, Business Source Complete, African Journals Online (AJOL), and Africa Index Medicus, alongside grey literature searching and citation tracking. We will include primary studies published in English (2015–2025) reporting qualitative, quantitative, or mixed-method findings. Screening and extraction will be conducted in duplicate, with discrepancies resolved by team members. Findings will be reported following PRISMA-ScR and synthesised narratively and thematically. Results will be disseminated through peer-reviewed publications, conferences, and webinars.

## Introduction

Climatic conditions across sub-Saharan Africa (SSA) have significant implications for agriculture, food security, and the economic livelihoods of about 70% of the region’s population [1,2]. Climate refers to the average weather conditions in a specific location over a long period of time, ranging from months to thousands or millions of years [3]. The World Meteorological Organization (WMO) uses a 30-year reference period to establish climatic averages. According to the Köppen Climate Classification, sub-Saharan Africa is predominantly characterised by equatorial climates, arid and semi-arid regions [4]. The climatic condition is increasingly being disrupted by climate change, with rising temperatures, altered rainfall patterns, frequent droughts, sea level rise and extreme heat in SSA [5,6]. These climatic changes also pose drastic challenges for sub-Saharan African populations, whose livelihoods, health, and well-being are closely tied to climate-sensitive sectors [7,8].

Among the populations most affected by climate change are informal outdoor workers in sub-Saharan Africa [9,10]. Informal outdoor workers are individuals engaged in income‑generating activities outside formal employment arrangements, often without social protection or occupational health and safety measures and are directly exposed to weather conditions such as solar radiation, humidity, and heat [11–13]. This group includes, but is not limited to, agricultural laborers, smallholder farmers, construction workers, street vendors, waste pickers, transport operators, and artisanal miners.

As climate change intensifies, rising temperatures and unpredictable weather reduce work capacity, disrupt daily work schedules, which may lead to income instability and economic losses among outdoor workers [10,14,15]. For instance, the 2024 report of the Lancet Countdown on health and climate change reported that heat exposure led to a record high loss of 512 billion potential labor hours in 2023, worth $835 billion in potential income losses globally [16]. Moreover, the prolonged exposure to harsh climatic conditions increases the risk of physical health problems, including heat-related illness and respiratory strain, and contributes to stress, anxiety, and declining mental well-being [7,11,17,18].

Beyond these climate-related health outcomes, the socio-economic context within which informal outdoor workers operate further shapes the extent of their vulnerability and capacity to respond [16]. Socioeconomic factors such as low-income levels and limited access to occupational health services influence outdoor workers’ capacity to cope with climate stressors [19]. These workers often labor without shade, cooling, or protective infrastructure, making them susceptible to heat stress, dehydration, and exhaustion as temperatures frequently rise, surpassing 35°C in cities like Accra and Nairobi [20,21]. Their livelihoods depend on daily earnings, yet erratic rainfall, floods, and droughts increasingly disrupt workdays and damage goods [22]. Without formal labor protections, savings, or access to social safety nets, these workers have limited capacity to adapt or recover, leaving them exposed to both health risks and long-term economic instability.

In this informal work context, it is likely that much of the adaptive response shifts to the individual rather than being supported structurally. A common pattern is the reliance on individual-level behavioural, attitudinal and cognitive adaptation strategies. These include wearing lightweight or breathable clothing, taking intermittent breaks, seeking shade where available, and increasing water intake to maintain hydration [23,24].

Despite growing evidence that climate change disproportionately affects informal outdoor workers’ health and work in sub-Saharan Africa, a scoping review to map the scope and nature of existing research remains unexplored. Some reviews have focused on outdoor workers broadly [10,11,14,15]. These studies have often grouped workers from both formal and informal outdoor occupational sectors together, overlooking the distinct nature of these work contexts [10,14,15]. This broad categorisation appears to limit understanding of sector-specific insights because informal outdoor workers, such as market vendors and street traders, operate under markedly different conditions compared to their formal outdoor counterparts. Formal outdoor workers, such as miners, police officers, traffic wardens, or construction workers, are often employed in formal organizations. Formal outdoor workers often have access to occupational safety measures, regulated work hours, and employer-provided health or social protections that may be absent in informal outdoor worker settings. The support they may receive from the formal employee may reduce the impacts of climate change on their work outcomes.

Consequently, the resources available to adapt to extreme heat events in developing countries (where informal employment is more common) differ markedly from those in more developed contexts. Hence, combining studies from both developed and developing country settings may limit understanding of how contextual differences shape outdoor workers’ vulnerability and adaptive responses.

Additionally, given that a large proportion of the population is engaged in informal outdoor work, it is essential to map the existing knowledge and adaptation practices within this group. Such synthesis is critical for identifying research gaps and guiding context-specific interventions that support climate resilience among informal outdoor workers.

The existing literature has mainly focused on the impact of climate change on occupational health among outdoor workers [10,11,15]. While this evidence is substantial, emerging findings suggest that climate change also influences other work-related outcomes such as job satisfaction and job insecurity [25–27]. From a theoretical perspective, the link between climate change and the mental wellbeing of workers in vulnerable industries can be understood through the Job Demands–Resources (JD–R) model [28,29]. This framework suggests that workers' wellbeing is shaped by the balance between job demands (aspects of work that require physical or psychological effort) and job resources (aspects that help meet their work goals and reduce job demands) [28]. Knowing what is available regarding other work outcomes, job demands, and resources affected by climate change among outdoor informal workers is also essential. A worker-centered scoping review is therefore imperative.

A scoping review was chosen because it aligns with the broad study’s aims of identifying and presenting the most current available information regarding the impacts of climate change on informal outdoor workers in SSA [30]. This approach helps summarise what is known, highlight areas needing further research, and determine whether a systematic review is warranted. In accordance with suggestions from Tricco et al. [30], this protocol was created to outline the purpose and methodology of the review. Publishing the protocol promotes transparency in the research process and helps prevent unnecessary duplication of work [31].

### Aim

This scoping review aims to identify and present the most current available information regarding the impacts of climate change on informal outdoor workers in Sub-Saharan Africa.

#### Specific objectives.

This scoping review seeks to:

1 Describe the range of physical health outcomes associated with climate-related exposures among informal outdoor workers.
2 Describe the range of socioeconomic and work-related outcomes associated with climate-related exposures among informal outdoor workers.
3 Present the climate-related mental health outcomes reported among informal outdoor workers in Sub-Saharan Africa.
4 Summarise the coping strategies documented among informal outdoor workers in response to climate-related exposures.

## Methods

The review whose development is planned in this protocol will be reported in accordance with the Preferred Reporting Items for Systematic Reviews and Meta-Analyses extension for Scoping Reviews (PRISMA-ScR) guideline.The development and planning of the review and protocol is also guided by the JBI [The Joanna Briggs Institute] approach and the PCC [Population [or participants]/Concept/Context] framework [31,32]. These two complementary guidelines, which share several authors, address methods and reporting, respectively [31,32]. The review will be carried out in accordance with this protocol, and details of any changes to this protocol will be reported in the final manuscript. S2 File presents the PRISMA-P checklist for this protocol.

### Identifying relevant studies

Authors will search for relevant documents across major public health databases, including Medline (Ovid), Global Health (Ovid), and Embase (Ovid), as well as multidisciplinary bibliographic databases such as Scopus and The Lens. PsycINFO (Ovid) and Business Source Complete (Ebsco) will be searched to capture research related to psychological and organisational aspects relevant to the review. Additionally, the research team will include Africa-Wide Information (Ebsco), African Journals Online (AJOL), and Africa Index Medicus in the search. By searching regional databases and The Lens, which provides extensive coverage of Low- and Middle-Income Countries (LMIC)-based journals, we aim to identify a wide range of relevant documents.

To capture documents not retrieved through electronic searches of journal article databases, authors will review grey literature in ProQuest Dissertations and the Council for the Development of Social Science Research in Africa (CODESRIA). Authors will also search organisational websites, such as WHO, and consult key scholars working on climate change impacts in SSA to identify additional reports and articles that may not be captured by other search strategies. This grey literature that presents primary data is likely to provide relevant evidence for this scoping review. To complement our searches in traditional bibliographic databases, we will also search Google Scholar and continue screening results until we encounter 20 consecutive entries that are not relevant.

Google Scholar has limitations that make it unsuitable as a primary source for systematic review searching [33,34]. Although it offers broad coverage, the content changes continuously, so identical searches can yield different results over time, undermining reproducibility and transparency requirements central to systematic review methodology. The platform only offers basic Boolean operators in search strings, which prevents the construction and documentation of search strategies needed to comprehensively identify all eligible studies [34]. However, Google Scholar can be used as a supplementary source [34,35]. To address the challenge of screening its low-precision, voluminous results, a practical stopping rule is recommended [36]. To balance comprehensiveness with efficiency, we deemed it adequate to continue title screening until Google Scholar relevance ranking presents 20 consecutive irrelevant entries.

Additionally, a reference check on included studies will be conducted to collect or monitor the citations of potentially applicable studies. This will be done using the Citation Chaser to identify forward citations of the included papers [37]. The reference lists of the documents included will also be screened to identify additional relevant sources that may not have appeared in the initial database searches via Citation Chaser. This backward citation tracking helps ensure a more comprehensive coverage of the literature by capturing studies that align with the review objectives but might be indexed differently or published in less commonly searched databases. By including this step, the review minimises the risk of missing key evidence and strengthens the overall completeness and reliability of the findings.

### Search strategies and process

PubMed searches were conducted initially to identify appropriate keywords and MeSH terms and develop the search strings. After testing the draft search to ensure it retrieved all known validation articles, search strings were developed for the other databases. The search string includes MeSH and keywords related to the concepts “climate change”, “outdoor”, “informal”, and “worker”. Truncation (‘*’), wildcards, and Boolean operators (AND/ OR) are used, as appropriate, to form the search strategy. Author KN, a research librarian at the Yale University School of Public Health, was consulted for the development of the search strings for all databases. An independent librarian peer-reviewed the PubMed search using the PRESS framework [38]. S1 File presents the detailed search strategies developed for the bibliographic databases that have already been searched: PubMed, Embase, Global Health, and PsycINFO. The detailed search strategies for these databases and the databases whose searches have not yet been conducted will all be presented in the future review manuscript.

### Study records

#### Data screening.

All retrieved studies’ bibliographic records will be imported into the Covidence Systematic Review Software, along with the predefined inclusion and exclusion criteria. Inclusion and exclusion criteria are presented in S1(Table 1). Covidence will be used to remove duplicate records.

**Table 1:** Inclusion and exclusion criteria.

Criteria	Inclusion	Exclusion
Population	Informal outdoor workers of all genders and age groups, including but not limited to farmers, construction workers, street vendors, waste pickers, transport workers, and other outdoor informal workers, will be included.	Primary research on indoor and formal outdoor workers, such as bank tellers, financial officers, IT professionals, software developers, bankers, laboratory scientists, and healthcare workers (e.g., nurses, pharmacists, lab technicians), and office workers (e.g., administrators, clerks, managers) will be excluded.
Concept	Primary studies that discuss or describe at least one change–related exposure (e.g., heat stress, extreme weather events, rainfall variability) and associated outcomes, including socioeconomic, work-related, physical health, mental health outcomes, and coping mechanisms, will be included.	Studies that focus solely on topics unrelated to climate change impacts or coping mechanisms will be excluded.
Context	Studies in any SSA country outlined by the United Nations M49 standard will be considered for inclusion [39]	Any studies conducted outside of SSA will not be considered, as the geographical focus is integral to the analysis of the subregion’s climate impacts and socioeconomic vulnerabilities. Studies that combine data from sub-Saharan African countries with data from other regions in a way that does not allow for the distinct extraction of SSA-specific data will be excluded.
Study designs	All empirical study designs, including qualitative, quantitative, and mixed methods approaches, will be eligible for inclusion.	Studies such as commentaries, opinion pieces, policy briefs, theoretical papers, reviews, meta-analyses, editorials, conference abstracts and white papers will be excluded from the review.
Time periods	Studies published from 2015 to 2025 will be eligible for inclusion. The review focuses on a limited year range to capture the most current knowledge available. Additionally, research on the nexus between climate change action and health and decent work in informal economies has gained momentum since the adoption of the Sustainable Development Goals (SDGs) in 2015.	Research published before 2015 will also be omitted to ensure that the review captures recent evidence on climate change impacts within the past decade.
Settings	Informal outdoor work settings in sub-Saharan Africa	Formal indoor and office-based work settings will be excluded.
Language	Full-text English-language studies will be considered for inclusion.	Studies published in other languages, such as French or Portuguese, will not be included in the analysis; however, their titles and abstracts will be noted and reported in a supplemental table, and future reviews may explore this literature further.

Two reviewers will pilot the screening procedure by independently screening the first 50 records at the title and abstract stage. Any records whose eligibility remains unclear after this stage will then be taken to full-text review during the screening phase. After establishing criteria, two independent reviewers will screen all remaining articles, with any disagreements resolved by the authors, CAB, ED, or YAB.

### Data extraction

#### Content.

Following article screening, authors will extract data on publication details, conceptualisation, methodology, and results. Publication details will include author names, year, journal, and funding source (where reported). For conceptualisation, authors will collect information on research questions or hypotheses, theoretical or conceptual frameworks, and variables studied (e.g., absenteeism, heat stress, occupational health, mental health outcomes, turnover intentions). Methodological data will cover study design (qualitative, quantitative, or mixed methodology), sampling and recruitment strategies (e.g., random sampling, snowball sampling), data collection methods (e.g., interviews, surveys, focus groups), analytical approaches (e.g., correlation, thematic analysis), and study setting (country, region). For results, we will extract participant information (e.g., sample size, sociodemographic characteristics such as age, income, and education) and study findings on climate change impacts and socioeconomic, work-related, physical, mental health outcomes, and coping mechanisms. Extracted findings will include thematic areas, participant quotes, and relevant statistical or descriptive measures. However, we will not draw conclusions from these findings, as scoping reviews focus on mapping the evidence.

#### Process.

To enable a systematic and coordinated approach to data extraction, we will use data extraction module 2 in the Covidence Systematic Review Software with questions for each extractor to enter the findings from the study. At least two authors will extract data from each article to ensure consistency. The two authors will meet to discuss and evaluate the extracted data for missing information or contradicting reports. Authors CBA, ED and YAB will meet with extractors to reach consensus on contradictory information if the primary reviewers cannot arrive at a decision.

#### Analyses and reporting.

The findings from the extracted data will be reported in accordance with PRISMA-ScR guidelines. Results will be presented in a table in line with the review questions. A narrative synthesis that highlights common themes addressing specific research questions will be reported.

### Ethical considerations

Since this study is a scoping review protocol, no ethical approval is required. However, adherence to ethical standards in reporting and data interpretation will be maintained throughout the review process.

## Discussion

This scoping review will systematically extract and synthesise outcomes associated with climate-related exposures among informal outdoor workers in SSA. To ensure alignment with review aims and conceptual clarity, the extracted data will be organised into four interconnected categories reflecting the worker-centred focus of this review. First, socioeconomic and work-related outcomes encompass changes in labour loss, job satisfaction, work interruptions, absenteeism, job insecurity and others. Second, mental health outcomes may capture conditions such as heat stress, heat strain, heat exhaustion, sleep disturbances, anxiety, psychological distress, burnout, occupational stress, post-traumatic stress symptoms, climate worry, eco-anxiety, and solastalgia. Third, physical health conditions will include acute and chronic bodily effects linked to climate exposure in occupational settings. Fourth, coping mechanisms will be extracted as adaptive behavioural and cognitive responses, such as adjusting work schedules, taking breaks, maintaining hydration, and using protective clothing. In addition to these predefined categories, any emergent outcomes that reflect worker-centred experiences or consequences of climate exposure will also be identified and extracted to ensure comprehensive mapping of the evidence. Synthesis of the extracted data will be primarily narrative and thematic.

A key strength of this review lies in its integration of climate change exposure with an organizational psychology theory, particularly the JD-R theory, to explain workers’ mental well-being. This perspective enables a more contextual understanding of how climate exposure intersects with job demands and resources in the informal outdoor work setting. Findings from this scoping review will be instrumental in shedding light on informal outdoor workers in SSA, a population that remains often underrepresented in occupational and climate–health research.

Additionally, this scoping review protocol adheres to the JBI methods guidelines and PRISMA-ScR reporting guidelines, ensuring a systematic, transparent, and reproducible approach to study identification, selection, and data extraction. The use of a clearly defined and replicable search strategy across multiple databases strengthens methodological rigor and enhances the reliability of the evidence mapping process. Moreover, this review will contribute to discussions on climate justice, ensuring that the vulnerabilities and needs of informal workers are not left out in the climate change adaptation strategies. Integrating climate adaptation measures into policies will be key to protecting informal outdoor workers from climate-induced economic and health shocks.

While this review aims to provide a comprehensive synthesis, the researchers acknowledge certain limitations. The inclusion of only primary studies may exclude valuable insights from editorials, commentaries, opinion pieces, and other reports not based on primary data. Another limitation is that only English-language studies will be included, which excludes evidence published in other languages from the review. In addition, authors will not conduct methodological quality appraisals of the included studies, which represents a potential limitation common to all scoping reviews. As methodological quality will not be formally appraised, conclusions about the strength of evidence for specific outcomes will be made cautiously; the review will primarily map outcome domains and evidence patterns rather than infer causality or quantify effect sizes. However, by employing rigorous scoping review methods (JBI methods guidelines) and reporting using PRISMA-ScR guidelines, the authors aim to ensure a transparent and high-quality synthesis of the available evidence.

Findings will be shared through peer-reviewed journals, conferences, and webinars targeting different audiences. This will help researchers, policymakers, and practitioners working on climate-related occupational health issues access and use the information. Also, presentations at local and international conferences and webinars will facilitate knowledge exchange with stakeholders.

## Supporting information

S1 File Search Strategies. (DOCX)

S2 File PRISMA P 2015 Checklist. (DOCX)
