# Supplementary material for: Socioeconomic, physical and mental health impacts of climate change among informal outdoor workers in sub-Saharan Africa: A scoping review protocol
Source: PLoS One. 2026 Mar 13;21(3):e0344943. doi: 10.1371/journal.pone.0344943 (PMC12987418; doi:10.1371/journal.pone.0344943)
Supplement: S1 File — (DOCX) [file pone.0344943.s001.docx]

**Search Strategies for Databases**

1. **PubMed - 929 results on 11/11/2025**

("laborer*"[Text Word] OR "labourer*"[Text Word] OR "vendor*"[Text Word] OR "hawker*"[Text Word] OR "trader*"[Text Word] OR "farmer*"[Text Word] OR "farmers"[MeSH Terms] OR "farmworker*"[Text Word] OR "market women*"[Text Word] OR "smallholder*"[Text Word] OR "small holder*"[Text Word] OR "waste picker*"[Text Word] OR "wastepicker*"[Text Word] OR "trash picker*"[Text Word] OR "trashpicker*"[Text Word] OR "carpenter*"[Text Word] OR "fishmonger*"[Text Word] OR "porter*"[Text Word] OR "kayayei"[Text Word] OR "plumber*"[Text Word] OR "harvester*"[Text Word] OR "subsistence"[Text Word] OR "gig worker*"[Text Word] OR "gig economy"[Text Word] OR "piecework*"[Text Word] OR "artisanal"[Text Word] OR "herder*"[Text Word] OR "shepherd*"[Text Word] OR "pastoralist*"[Text Word] OR "recycler*"[Text Word] OR "recycling worker*"[Text Word] OR "waste handler*"[Text Word] OR "miner"[Text Word] OR "miners"[Text Word] OR "galamsey"[Text Word] OR (("worker*"[Text Word] OR "work"[Text Word] OR "workplace*"[Text Word] OR "occupational exposure"[MeSH Terms] OR "occupational groups"[MeSH Terms:noexp] OR "occupations"[MeSH Terms] OR "employment"[MeSH Terms:noexp] OR "workplace"[MeSH Terms] OR "occupational"[Text Word]) AND ("outdoor"[Text Word] OR "informal"[Text Word] OR "migrant*"[Text Word] OR "immigrant*"[Text Word] OR "undocumented"[Text Word] OR "semi-formal"[Text Word] OR "semiformal"[Text Word] OR "refugee*"[Text Word] OR "open-air"[Text Word] OR "casual"[Text Word]))) AND ("angola*"[Text Word] OR "botswana*"[Text Word] OR "lesotho*"[Text Word] OR "malawi*"[Text Word] OR "mozambiq*"[Text Word] OR "namibia*"[Text Word] OR "Swaziland"[Text Word] OR "zambia*"[Text Word] OR "Zimbabwe"[Text Word] OR "Zulu"[Text Word] OR "Tsonga"[Text Word] OR "Xhosa"[Text Word] OR "Swazi"[Text Word] OR "Ndebele"[Text Word] OR "Tswana"[Text Word] OR "Sotho"[Text Word] OR "shona people"[Text Word] OR "Ovimbundu"[Text Word] OR "Chaga"[Text Word] OR "Sukuma"[Text Word] OR "Pretoria"[Text Word] OR "cape town"[Text Word] OR "Johannesburg"[Text Word] OR "Durban"[Text Word] OR "port elizabeth"[Text Word] OR "Bloemfontein"[Text Word] OR "Windhoek"[Text Word] OR "Maseru"[Text Word] OR ("Kimberley"[Text Word] NOT "Australia"[Text Word]) OR "Soweto"[Text Word] OR "Polokwane"[Text Word] OR "Limpopo"[Text Word] OR "Rustenburg"[Text Word] OR "Mahikeng"[Text Word] OR "Stellenbosch"[Text Word] OR "Paarl"[Text Word] OR "Gaborone"[Text Word] OR "Luanda"[Text Word] OR "Cabinda"[Text Word] OR "Huambo"[Text Word] OR "Lubango"[Text Word] OR "Malanje"[Text Word] OR "Lobito"[Text Word] OR "Lilongwe"[Text Word] OR "Blantyre"[Text Word] OR "Mzuzu"[Text Word] OR "Maputo"[Text Word] OR "Matola"[Text Word] OR "Beira"[Text Word] OR "Nampula"[Text Word] OR "Chimoio"[Text Word] OR "Nacala"[Text Word] OR "Quelimane"[Text Word] OR "Lusaka"[Text Word] OR "Kitwe"[Text Word] OR "Ndola"[Text Word] OR "Kabwe"[Text Word] OR "Copperbelt"[Text Word] OR "Harare"[Text Word] OR "Bulawayo"[Text Word] OR "Chitungwiza"[Text Word] OR "Mutare"[Text Word] OR "Masvingo"[Text Word] OR "Manicaland"[Text Word] OR "sub saharan africa*"[Text Word] OR "subsaharan africa*"[Text Word] OR "sub-saharan africa"[Title/Abstract:~3] OR "subsaharan africa"[Title/Abstract:~3] OR "Africa South of the Sahara"[MeSH Terms] OR "africa"[MeSH Terms:noexp] OR "africa*"[Text Word] OR "Angola"[Text Word] OR "Benin"[Text Word] OR "Botswana"[Text Word] OR "Burkina Faso"[Text Word] OR "Burundi"[Text Word] OR "Cabo Verde"[Text Word] OR "Cape Verde"[Text Word] OR "Cameroon"[Text Word] OR "central african republic"[Text Word] OR "Chad"[Text Word] OR "Comoros"[Text Word] OR "Congo"[Text Word] OR "Cote d'Ivoire"[Text Word] OR "Ivory Coast"[Text Word] OR "Djibouti"[Text Word] OR "Equatorial Guinea"[Text Word] OR "Eritrea"[Text Word] OR "Eswatini"[Text Word] OR "Ethiopia"[Text Word] OR "Gabon"[Text Word] OR "Gambia"[Text Word] OR "Ghana"[Text Word] OR "Guinea"[Text Word] OR "Guinea-Bissau"[Text Word] OR "Kenya"[Text Word] OR "Lesotho"[Text Word] OR "Liberia"[Text Word] OR "Madagascar"[Text Word] OR "Malawi"[Text Word] OR "Mali"[Text Word] OR "Mauritania"[Text Word] OR "Mauritius"[Text Word] OR "Mozambique"[Text Word] OR "Namibia"[Text Word] OR "Niger"[Text Word] OR "Nigeria"[Text Word] OR "Rwanda"[Text Word] OR "Sao Tome"[Text Word] OR "Principe"[Text Word] OR "Senegal"[Text Word] OR "Seychelles"[Text Word] OR "Sierra Leone"[Text Word] OR "Somalia"[Text Word] OR "South Africa"[Text Word] OR "Sub Saharan"[Text Word] OR "Sudan"[Text Word] OR "Tanzania"[Text Word] OR "Togo"[Text Word] OR "Tunisia"[Text Word] OR "Uganda"[Text Word] OR "Zambia"[Text Word] OR "Zimbabwe"[Text Word] OR "Swaziland"[Text Word]) AND ("climate change"[MeSH Terms] OR (1993/01/01:2009/12/31[Date - MeSH] AND "climate"[MeSH Terms:noexp]) OR "extreme heat"[MeSH Terms] OR "climate change"[Text Word] OR "global warming"[Text Word] OR "extreme heat"[Text Word] OR "heatwave*"[Text Word] OR "heat wave*"[Text Word] OR "wet bulb temperature*"[Text Word] OR "hot weather"[Text Word] OR "warmer weather"[Text Word] OR "warm weather"[Text Word] OR "hot temperature"[MeSH Terms] OR "droughts"[MeSH Terms] OR "drought*"[Text Word] OR "climate event*"[Text Word] OR "climatic event*"[Text Word] OR "weather event*"[Text Word] OR "floods"[MeSH Terms] OR "flood*"[Text Word] OR "rainfall"[Text Word] OR "bushfire*"[Text Word] OR "bush fire*"[Text Word] OR "wildfire*"[Text Word] OR "wild fire*"[Text Word] OR "wildfires"[MeSH Terms] OR "heat index"[Text Word] OR "air temperature*"[Text Word] OR "sunheat"[Text Word] OR "sun heat"[Text Word]) AND 2015/01/01:2025/12/31[Date - Publication]

1. **Embase via OVID platform on 16^th^ January,2026**

| # | Query | Results from 16 Jan 2026 |
| --- | --- | --- |
| 1 | [2026-01-16 Embase translation of the PubMed search for Sylvia's climate change outdoor worker project] | 0 |
| 2 | [part 1 workers] | 0 |
| 3 | exp outdoor worker/ | 35,065 |
| 4 | migrant worker/ | 2,451 |
| 5 | beekeeper/ or blue collar worker/ or carpenter/ or construction worker/ or fisherman/ or gardener/ or greenhouse worker/ or miner/ or waste picker/ | 10,456 |
| 6 | ("laborer*" or "labourer*" or "vendor*" or "hawker*" or "trader*" or "farmer*" or "farmworker*" or "market women*" or "smallholder*" or "small holder*" or "waste picker*" or "wastepicker*" or "trash picker*" or "trashpicker*" or "carpenter*" or "fishmonger*" or "porter*" or "kayayei" or "plumber*" or "harvester*" or "subsistence" or "gig worker*" or "gig economy" or "piecework*" or "artisanal" or "herder*" or "shepherd*" or "pastoralist*" or "recycler*" or "recycling worker*" or "waste handler*" or "miner" or "miners" or "galamsey").mp. | 99,307 |
| 7 | ("outdoor" or "informal" or "migrant*" or "immigrant*" or "undocumented" or "semi-formal" or "semiformal" or "refugee*" or "open-air" or "casual").mp. | 198,042 |
| 8 | (worker* or work or workplace* or occupational).mp. | 2,630,249 |
| 9 | exp worker/ | 68,118 |
| 10 | occupational health/ or exp "occupation and occupation related phenomena"/ | 1,103,350 |
| 11 | 7 and (8 or 9 or 10) | 46,103 |
| 12 | 3 or 4 or 5 or 6 | 120,782 |
| 13 | 11 or 12 | 161,561 |
| 14 | [summation of the outdoor worker concept] | 0 |
| 15 | [part 2: SSA] | 0 |
| 16 | ("angola*" or "botswana*" or "lesotho*" or "malawi*" or "mozambiq*" or "namibia*" or "Swaziland" or "zambia*" or "Zimbabwe" or "Zulu" or "Tsonga" or "Xhosa" or "Swazi" or "Ndebele" or "Tswana" or "Sotho" or "shona people" or "Ovimbundu" or "Chaga" or "Sukuma" or "Pretoria" or "cape town" or "Johannesburg" or "Durban" or "port elizabeth" or "Bloemfontein" or "Windhoek" or "Maseru" or ("Kimberley" not "Australia") or "Soweto" or "Polokwane" or "Limpopo" or "Rustenburg" or "Mahikeng" or "Stellenbosch" or "Paarl" or "Gaborone" or "Luanda" or "Cabinda" or "Huambo" or "Lubango" or "Malanje" or "Lobito" or "Lilongwe" or "Blantyre" or "Mzuzu" or "Maputo" or "Matola" or "Beira" or "Nampula" or "Chimoio" or "Nacala" or "Quelimane" or "Lusaka" or "Kitwe" or "Ndola" or "Kabwe" or "Copperbelt" or "Harare" or "Bulawayo" or "Chitungwiza" or "Mutare" or "Masvingo" or "Manicaland" or "sub saharan africa*" or "subsaharan africa*" or "africa*" or "Angola" or "Benin" or "Botswana" or "Burkina Faso" or "Burundi" or "Cabo Verde" or "Cape Verde" or "Cameroon" or "central african republic" or "Chad" or "Comoros" or "Congo" or "Cote d'Ivoire" or "Ivory Coast" or "Djibouti" or "Equatorial Guinea" or "Eritrea" or "Eswatini" or "Ethiopia" or "Gabon" or "Gambia" or "Ghana" or "Guinea" or "Guinea-Bissau" or "Kenya" or "Lesotho" or "Liberia" or "Madagascar" or "Malawi" or "Mali" or "Mauritania" or "Mauritius" or "Mozambique" or "Namibia" or "Niger" or "Nigeria" or "Rwanda" or "Sao Tome" or "Principe" or "Senegal" or "Seychelles" or "Sierra Leone" or "Somalia" or "South Africa" or "Sub Saharan" or "Sudan" or "Tanzania" or "Togo" or "Tunisia" or "Uganda" or "Zambia" or "Zimbabwe" or "Swaziland").mp. | 961,770 |
| 17 | ((sub-saharan adj3 africa*) or (subsaharan adj3 africa*)).mp. | 52,685 |
| 18 | exp "Africa south of the Sahara"/ | 368,655 |
| 19 | 16 or 17 or 18 [SSA concept] | 962,016 |
| 20 | [part 3: climate change] | 0 |
| 21 | exp climate change/ or climate change mitigation/ or climate change vulnerability/ | 88,713 |
| 22 | exp heat wave/ | 4,186 |
| 23 | ("climate change" or "global warming" or "extreme heat" or "heatwave*" or "heat wave*" or "wet bulb temperature*" or "hot weather" or "warmer weather" or "warm weather" or "drought*" or "climate event*" or "climatic event*" or "weather event*" or "flood*" or "rainfall" or "bushfire*" or "bush fire*" or "wildfire*" or "wild fire*" or "heat index" or "air temperature*" or "sunheat" or "sun heat").mp. | 230,221 |
| 24 | 21 or 22 or 23 [climate summation] | 230,807 |
| 25 | [the combination!] | 0 |
| 26 | 13 and 19 and 24 | 1,161 |
| 27 | limit 26 to yr=2015-2026 | 884 |

1. **Global Health via Ovid platform on 17th December 2025**

| **#** | **Query** | **Results from 17 Dec 2025** |
| --- | --- | --- |
| 1 | [2025-12-08 Global Health translation of the PubMed search for Sylvia's climate change outdoor worker project] | 0 |
| 2 | [part 1 workers] | 0 |
| 3 | auxiliary workers/ or basket weavers/ or beekeepers/ or construction workers/ or drivers/ or exp farm workers/ or farmers/ or fertilizer workers/ or fishermen/ or food handlers/ or growers/ or market traders/ or merchants/ or milkers/ or miners/ or park rangers/ or peasant workers/ or stockmen/ or stone workers/ | 37,567 |
| 4 | ("laborer*" or "labourer*" or "vendor*" or "hawker*" or "trader*" or "farmer*" or "farmworker*" or "market women*" or "smallholder*" or "small holder*" or "waste picker*" or "wastepicker*" or "trash picker*" or "trashpicker*" or "carpenter*" or "fishmonger*" or "porter*" or "kayayei" or "plumber*" or "harvester*" or "subsistence" or "gig worker*" or "gig economy" or "piecework*" or "artisanal" or "herder*" or "shepherd*" or "pastoralist*" or "recycler*" or "recycling worker*" or "waste handler*" or "miner" or "miners" or "galamsey").mp. | 71,829 |
| 5 | ("outdoor" or "informal" or "migrant*" or "immigrant*" or "undocumented" or "semi-formal" or "semiformal" or "refugee*" or "open-air" or "casual").mp. | 78,629 |
| 6 | (worker* or work or workplace* or occupational).mp. | 567,298 |
| 7 | workers/ or personnel/ | 82,911 |
| 8 | occupational health/ or exp occupational hazards/ or safety at work/ or work/ or working conditions/ | 91,336 |
| 9 | 3 or 4 or farming/ [implicitly outdoor jobs] | 88,770 |
| 10 | 5 and (6 or 7 or 8) [work in general, in potential outdoor settings] | 18,628 |
| 11 | 9 or 10 [part 1 summation: workers] | 104,627 |
| 12 | [part 2: SSA] | 0 |
| 13 | ("angola*" or "botswana*" or "lesotho*" or "malawi*" or "mozambiq*" or "namibia*" or "Swaziland" or "zambia*" or "Zimbabwe" or "Zulu" or "Tsonga" or "Xhosa" or "Swazi" or "Ndebele" or "Tswana" or "Sotho" or "shona people" or "Ovimbundu" or "Chaga" or "Sukuma" or "Pretoria" or "cape town" or "Johannesburg" or "Durban" or "port elizabeth" or "Bloemfontein" or "Windhoek" or "Maseru" or ("Kimberley" not "Australia") or "Soweto" or "Polokwane" or "Limpopo" or "Rustenburg" or "Mahikeng" or "Stellenbosch" or "Paarl" or "Gaborone" or "Luanda" or "Cabinda" or "Huambo" or "Lubango" or "Malanje" or "Lobito" or "Lilongwe" or "Blantyre" or "Mzuzu" or "Maputo" or "Matola" or "Beira" or "Nampula" or "Chimoio" or "Nacala" or "Quelimane" or "Lusaka" or "Kitwe" or "Ndola" or "Kabwe" or "Copperbelt" or "Harare" or "Bulawayo" or "Chitungwiza" or "Mutare" or "Masvingo" or "Manicaland" or "sub saharan africa*" or "subsaharan africa*" or "africa*" or "Angola" or "Benin" or "Botswana" or "Burkina Faso" or "Burundi" or "Cabo Verde" or "Cape Verde" or "Cameroon" or "central african republic" or "Chad" or "Comoros" or "Congo" or "Cote d'Ivoire" or "Ivory Coast" or "Djibouti" or "Equatorial Guinea" or "Eritrea" or "Eswatini" or "Ethiopia" or "Gabon" or "Gambia" or "Ghana" or "Guinea" or "Guinea-Bissau" or "Kenya" or "Lesotho" or "Liberia" or "Madagascar" or "Malawi" or "Mali" or "Mauritania" or "Mauritius" or "Mozambique" or "Namibia" or "Niger" or "Nigeria" or "Rwanda" or "Sao Tome" or "Principe" or "Senegal" or "Seychelles" or "Sierra Leone" or "Somalia" or "South Africa" or "Sub Saharan" or "Sudan" or "Tanzania" or "Togo" or "Tunisia" or "Uganda" or "Zambia" or "Zimbabwe" or "Swaziland").mp. | 703,123 |
| 14 | ((sub-saharan adj3 africa*) or (subsaharan adj3 africa*)).mp. | 497,423 |
| 15 | exp Southern Africa/ | 115,510 |
| 16 | exp west africa/ | 173,997 |
| 17 | exp central africa/ | 44,507 |
| 18 | exp east africa/ | 181,506 |
| 19 | tunisia/ | 9,504 |
| 20 | exp comoros/ | 819 |
| 21 | exp mauritius/ | 1,797 |
| 22 | "africa south of sahara"/ | 493,534 |
| 23 | "sub saharan".mp. | 43,720 |
| 24 | [subsaharan.mp](http://subsaharan.mp/). | 493,124 |
| 25 | or/13-24 [summation of part 2, SSA] | 703,125 |
| 26 | [part 3, climate change] | 0 |
| 27 | ("climate change" or "global warming" or "extreme heat" or "heatwave*" or "heat wave*" or "wet bulb temperature*" or "hot weather" or "warmer weather" or "warm weather" or "drought*" or "climate event*" or "climatic event*" or "weather event*" or "flood*" or "rainfall" or "bushfire*" or "bush fire*" or "wildfire*" or "wild fire*" or "heat index" or "air temperature*" or "sunheat" or "sun heat").mp. | 70,096 |
| 28 | exp climate change/ | 22,106 |
| 29 | exp environmental temperature/ | 13,732 |
| 30 | heat/ | 5,899 |
| 31 | temperature/ or night temperature/ | 84,959 |
| 32 | natural disasters/ or floods/ | 9,541 |
| 33 | drought/ | 5,053 |
| 34 | or/27-33 [summation of part 3, climate change] | 160,452 |
| 35 | 11 and 25 and 34 | 2,092 |
| 36 | limit 35 to yr=2015-2026 | 1,009 |

1. **PsycINFO Via Ovid platform on 8 December, 2025**

| **#** | **Query** | **Results from 8 Dec 2025** |
| --- | --- | --- |
| 1 | [2025-12-08 PsycINFO translation of the PubMed search for Sylvia's climate change outdoor worker project] | 0 |
| 2 | [part 1 workers] | 0 |
| 3 | exp agricultural workers/ or exp migrant workers/ | 3,407 |
| 4 | ("laborer*" or "labourer*" or "vendor*" or "hawker*" or "trader*" or "farmer*" or "farmworker*" or "market women*" or "smallholder*" or "small holder*" or "waste picker*" or "wastepicker*" or "trash picker*" or "trashpicker*" or "carpenter*" or "fishmonger*" or "porter*" or "kayayei" or "plumber*" or "harvester*" or "subsistence" or "gig worker*" or "gig economy" or "piecework*" or "artisanal" or "herder*" or "shepherd*" or "pastoralist*" or "recycler*" or "recycling worker*" or "waste handler*" or "miner" or "miners" or "galamsey").mp. | 16,994 |
| 5 | ("outdoor" or "informal" or "migrant*" or "immigrant*" or "undocumented" or "semi-formal" or "semiformal" or "refugee*" or "open-air" or "casual").mp. | 111,492 |
| 6 | (worker* or work or workplace* or occupational).mp. | 812,305 |
| 7 | exp personnel/ | 739,973 |
| 8 | occupations/ or exp employment status/ or exp job characteristics/ or occupational exposure/ or exp occupational health/ or occupational safety/ or occupational status/ or exp occupational stress/ or working women/ | 98,863 |
| 9 | 5 and (6 or 7 or 8) | 38,428 |
| 10 | 4 or 9 | 54,432 |
| 11 | [above is the worker summation] | 0 |
| 12 | [part 2: SSA] | 0 |
| 13 | ("angola*" or "botswana*" or "lesotho*" or "malawi*" or "mozambiq*" or "namibia*" or "Swaziland" or "zambia*" or "Zimbabwe" or "Zulu" or "Tsonga" or "Xhosa" or "Swazi" or "Ndebele" or "Tswana" or "Sotho" or "shona people" or "Ovimbundu" or "Chaga" or "Sukuma" or "Pretoria" or "cape town" or "Johannesburg" or "Durban" or "port elizabeth" or "Bloemfontein" or "Windhoek" or "Maseru" or ("Kimberley" not "Australia") or "Soweto" or "Polokwane" or "Limpopo" or "Rustenburg" or "Mahikeng" or "Stellenbosch" or "Paarl" or "Gaborone" or "Luanda" or "Cabinda" or "Huambo" or "Lubango" or "Malanje" or "Lobito" or "Lilongwe" or "Blantyre" or "Mzuzu" or "Maputo" or "Matola" or "Beira" or "Nampula" or "Chimoio" or "Nacala" or "Quelimane" or "Lusaka" or "Kitwe" or "Ndola" or "Kabwe" or "Copperbelt" or "Harare" or "Bulawayo" or "Chitungwiza" or "Mutare" or "Masvingo" or "Manicaland" or "sub saharan africa*" or "subsaharan africa*" or "africa*" or "Angola" or "Benin" or "Botswana" or "Burkina Faso" or "Burundi" or "Cabo Verde" or "Cape Verde" or "Cameroon" or "central african republic" or "Chad" or "Comoros" or "Congo" or "Cote d'Ivoire" or "Ivory Coast" or "Djibouti" or "Equatorial Guinea" or "Eritrea" or "Eswatini" or "Ethiopia" or "Gabon" or "Gambia" or "Ghana" or "Guinea" or "Guinea-Bissau" or "Kenya" or "Lesotho" or "Liberia" or "Madagascar" or "Malawi" or "Mali" or "Mauritania" or "Mauritius" or "Mozambique" or "Namibia" or "Niger" or "Nigeria" or "Rwanda" or "Sao Tome" or "Principe" or "Senegal" or "Seychelles" or "Sierra Leone" or "Somalia" or "South Africa" or "Sub Saharan" or "Sudan" or "Tanzania" or "Togo" or "Tunisia" or "Uganda" or "Zambia" or "Zimbabwe" or "Swaziland").mp. | 152,166 |
| 14 | ((sub-saharan adj3 africa*) or (subsaharan adj3 africa*)).mp. | 6,255 |
| 15 | 13 or 14 | 152,166 |
| 16 | [part 3: climate change] | 0 |
| 17 | climate change/ or global warming/ or climate anxiety/ or climate change attitudes/ or extreme weather/ or heat effects/ or exp temperature effects/ or thermal acclimatization/ | 11,158 |
| 18 | seasonal variations/ | 3,768 |
| 19 | ("climate change" or "global warming" or "extreme heat" or "heatwave*" or "heat wave*" or "wet bulb temperature*" or "hot weather" or "warmer weather" or "warm weather" or "drought*" or "climate event*" or "climatic event*" or "weather event*" or "flood*" or "rainfall" or "bushfire*" or "bush fire*" or "wildfire*" or "wild fire*" or "heat index" or "air temperature*" or "sunheat" or "sun heat").mp. | 13,472 |
| 20 | 17 or 18 or 19 | 22,502 |
| 21 | [the combination] | 0 |
| 22 | 10 and 15 and 20 | 85 |
| 23 | limit 22 to yr=2015-2025 | 55 |

Note: the December 2025 and January 2026 iterations of the search strategy retrieved documents about Tunisia, but those documents are not eligible because they don't meet the criterion of geographic location in Sub-Saharan Africa. In later iterations of the search, the term Tunisia was not used. This initial mistake slightly decreased the specificity of the search strategy, but it had no impact on the sensitivity of the search strategy.
